# Left and right ventricular functions may be impaired in children diagnosed with subclinical hypothyroidism

**DOI:** 10.1038/s41598-020-76327-4

**Published:** 2020-11-12

**Authors:** Alper Akın, Edip Unal, Ruken Yildirim, Mehmet Ture, Hasan Balik, Yusuf Kenan Haspolat

**Affiliations:** 1 Department of Pediatric Cardiology, Dicle University Faculty of Medicine, Sur, Diyarbakır, Turkey; 2 Department of Pediatric Endocrinology, Dicle University Faculty of Medicine, Diyarbakır, Turkey

**Keywords:** Cardiovascular biology, Thyroid diseases

## Abstract

Subclinical hypothyroidism (SH) may influence both ventricular functions. The aim of this study was to evaluation the findings of Tissue Doppler Imaging (TDI) and other echocardiography modalities in children with SH. We compared left ventricular mass index (LVMI) and TDI parameters of patients with SH and children with euthyroidism. Subclinical hypothyroidism was diagnosed when thyroid stimulating hormone level was higher than the reference value of the laboratory (> 4.2 mIU/L) and free thyroxine level was in normal range. The study included a group of 35 patients with SH and a control group of 38 children with euthyroidism (mean age was 7.6 ± 3.5 years and 9.0 ± 2.4 years, respectively). LVMI was significantly higher in the patient group (p = 0.005). TDI parameters including mitral septal ejection time was lower (p = 0.003) and mitral septal myocardial performance index was higher (p = 0.009) in the patient group. Right ventricular TDI revealed that tricuspid lateral E/Ea and tricuspid septal E/Ea were higher (p = 0.015 and p = 0.024, respectively) and tricuspid septal Ea/Aa and ejection time were lower (p = 0.018 and p = 0.017, respectively) in the patient group. SH may lead to increase LVMI. Left ventricular systolic and diastolic TDI parameters (lower mitral septal ejection time, higher mitral septal myocardial performance index) as well as right ventricular systolic (lower tricuspid septal ejection time) and diastolic (higher tricuspid septal and lateral E/Ea, lower tricuspid septal Ea/Ea) functions may be also impaired in children with subclinical hypothyroidism. TDI is a useful method used for the assessment of the effect of SH on cardiac functions.

## Introduction

Subclinical hypothyroidism is diagnosed when serum thyroid stimulating hormone level is increased and free thyroxine level is within normal reference^1^. Subclinical hypothyroidism has a prevalence of 3–18% of in adults^2^. Although, the prevalence of subclinical hypothyroidism in children remains unknown, it is reported that subclinical hypothyroidism is less frequent in children compared to adults^3,4^. Although numerous factors have been blamed in the etiology of subclinical hypothyroidism, autoimmune disorders of the thyroid gland are considered to be the most common cause of subclinical hypothyroidism both in children and adults^5^.

Most studies investing the effects of subclinical hypothyroidism have mostly been conducted in adults and there is a paucity of data regarding subclinical hypothyroidism in children. Subclinical hypothyroidism has been shown to influence the neuromuscular system, carbohydrate mechanism, and cognitive functions^6–8^. Nevertheless, subclinical hypothyroidism mainly attacks the cardiovascular system, mostly leading to left and right ventricular systolic and diastolic dysfunction, heart failure, coronary heart disease, vascular dysfunction with increased vascular stiffness and endothelial dysfunction, and increased left ventricular mass^9–13^^.^

Tissue Doppler imaging findings have been shown to be important in the subclinical hypothyroidism; however, most of these findings have been reported in adult studies^10,14–16^. The aim of this study was to compare the findings of Tissue Doppler Imaging and other echocardiography modalities (Two-dimensional and M-mode echocardiography) in children with subclinical hypothyroidism and control groups.

## Methods

The study included 35 children diagnosed with subclinical hypothyroidism at Dicle University Medical School Department of Pediatric Endocrinology between September 2016 and February 2017 and 38 healthy euthyroid children with normal thyroid functions. Normal reference values of our laboratory where the study was conducted were as follows: thyroid stimulating hormone 0.27–4.2 mIU/L, serum-free triiodothyronine 3.69–9.85 pmol/L, and free thyroxine 12–22.8 pmol/L. The diagnosis of subclinical hypothyroidism was established based on an increased thyroid stimulating hormone level (4.2–20 mIU/L) and normal serum-free triiodothyronine and free thyroxine levels measured in two morning fasting blood samples that were obtained at a minimum of a 15-day interval. Measurement of serum thyroid stimulating hormone, serum-free triiodothyronine and free thyroxine levels was performed by electrochemiluminescence immunoassay (ECLIA) method with a Cobos e601 analyzer (Roche HITACHI, Germany).

The children with SH were referred to pediatric endocrinology clinic by their physicians because of elevated serum TSH concentrations detected during check-up. No signs of hypertension, liver and kidney dysfunctions, lung disease, systemic infection, or chronic diseases were detected in any subject in both groups. Moreover, no subject was using medication during the study. The study excluded patients with obesity, low birth weight, diabetes mellitus, and a history of drug use. Electrocardiogram indicated normal sinus rhythm in all the subjects. In both groups’ Transthoracic Echocardiographic examinations, the subjects that were detected with any pathology that could affect Tissue Doppler Imaging, such as valvular heart disease and congenital heart defect, were excluded from the study.

All the echocardiographic examinations were performed by an experienced pediatric cardiologist who was blinded to the clinical and biochemical characteristics of the patients. A written consent was received from the parents of each subject. The study was approved by the local ethics committee.

Transthorasic echocardiographic examinations was performed using a 2–4 MHz phased-array transducer (Vivid S5; GE, Horten, Norway). Transducers of 2.5 or 3.5 MHz were used. Color flow and 2D Doppler echocardiography were performed at the apical four chamber, parasternal long axis, parasternal short axis, subcostal and at the suprasternal positions. M-mode echocardiography was performed at the parasternal long axis for the measurement of left ventricle end-diastolic diameter and left ventricle end-systolic diameter, fractional shortening, and ejection fraction. Left ventricle mass index was calculated by using the formula proposed by Devereux’s et al.^14^. Using conventional Doppler transthoracic echocardiographic examinations, mitral and tricuspid early diastolic E wave (m/s), late diastolic A wave (m/s), and E/A ratio were determined and evaluated in accordance with the American Society of Echocardiography guidelines^17^.

For the measurement of tissue Doppler, the pulse wave sample was positioned on the mitral lateral, mitral septal, tricuspid lateral and at the junction of the septal cusp of the tricuspid valve with the ventricle. For the measurement of interventricular septum, the pulse wave sample was placed parallel to the septum and was positioned at its upper 1/3 part. Tissue Doppler Imaging was used to determine early diastolic velocity (E_a,_ m/s), late diastolic velocity (A_a_, m/s), systolic velocity (S_a_, m/s), acceleration time (ms), myocardial performance index, isovolumic contraction time (IVCT, ms), deceleration time (ms), ejection time (ejection time, ms) of the mitral valve, isovolumic relaxation time (IVRT, ms), tricuspid valve, and interventricular septum.

### Statistics

Statistical analysis was performed by IBM SPSS 21.0 for Windows (SPSS Inc. Co., Chicago, IL, USA). Quantitative variables were expressed as mean ± standard deviation (SD) and categorical variables as count and percentage (%). Comparison of binary variables was performed by using independent samples *t*-test for normally distributed variables and Mann Whitney U test for non-normally distributed variables. Comparison of qualitative variables was performed by chi-square test. Pearson Correlation Coefficient was used for the analysis of the relationship among numerical variables. The hypotheses were two-tailed and a *p* value of ≤ 0.05 was considered significant.

### Ethical approval

The authors assert that all procedures contributing to this study comply with the ethical standards of the relevant national guidelines on human experimentation and with the Helsinki Declaration of 1975, as revised in 2008, and has been approved by the institutional committees of Dicle University.

### Informed consent

Informed consent was given by the participants or by their legal guardians.

### Conference presentation

This study was presented at “52nd Annual Meeting of the Association for European Paediatric and Congenital Cardiology (in May 09–12, 2018, at Ahtens-Greece)” as a poster presentation.

## Results

The study included a group of 35 (19 male, 16 female) patients with subclinical hypothyroidism and a control group of 38 healthy (20 male, 18 female) children with euthyroidism. Mean age was 7.6 ± 3.5 years in the patient group and 9.0 ± 2.4 years in the control group. No significant difference was found between the groups in terms of age, gender, body weight, and body mass index. Thyroid stimulating hormone was significantly higher (*p* < 0.001) and serum-free triiodothyronine and free thyroxine levels were similar compared to the control group. Of the 35 patients, 31 of them had a thyroid stimulating hormone of 5–10 mIU/L and 4 had a thyroid stimulating hormone of ≥ 10 mIU/L. Table 1 presents the demographic and laboratory characteristics of the subjects in both groups.

**Table 1:** Demographic and laboratory characteristic of the study groups.

	SH group (n = 35)	Control group (n = 38)	*p* value
Gender (female/male)	19/16	20/18	0.887
Age (year)	7.6 ± 3.5 6.5 (3.5–15.0)	9.0 ± 2.4 8.7 (4.5–15.0)	0.232
Weight (kg)	25.4 ± 11.8 21.0 (12.8–63.0)	29.3 ± 10.3 27.7 (16.0–57.0)	0.141
Height (cm)	121.2 ± 19.44	128.0 ± 13.33	0.076
BMI (kg/m^2^)	16.4 ± 2.4 15.7 (13.7–25.0)	16.6 ± 2.8 15.6 (12.2–23.5)	0.899
Systolic blood pressure (mmHg)	104.5 ± 5.5	106.3 ± 6.7	0.241
Diastolic blood pressure (mmHg)	63.3 ± 5.6	65.8 ± 7.6	0.150
Heart rate (beats a minute)	92.0 ± 16.4	87.0 ± 15.1	0.182
TSH (mIU/L)	7.5 ± 2.3 6.7 (5.0–14.5)	2.4 ± 0.7 2.3 (1.1–3.9)	< 0.001
fT4 (pmol/L)	16.2 ± 1.8 16.6 (12.6–19.0)	16.9 ± 1.9 16.4 (13.2–21.4)	0.164
fT3 (pmol/L)	6.6 ± 0.6 6.7 (5.6–7.2)	6.7 ± 0.7 6.6 (4.6–8.7)	0.655

Data are given as mean ± SD, and median (range).

*BMI* body mass index, *fT3* free triiodothyronine, *fT4* free thyroxine, *SH* subclinical hypothyroidism, *TSH* thyroid-stimulating hormone.

Left ventricular mass index was significantly higher in the patient group compared to the control group (*p* = 0.005). However, left ventricle end-diastolic diameter, fractional shortening, and ejection fraction established no significant difference between the groups. Left ventricular end-systolic diameter was lower in the patient group compared to the control group (*p* = 0.023). Table 2 presents M-Mode echocardiographic measurements of the two groups.

**Table 2:** M-Mode echocardiographic measurements of the study groups.

	SH group (n = 35)	Control group (n = 38)	*p* value
LVEDd (cm)	3.4 ± 0.5	3.6 ± 0.3	0.139
LVESd (cm)	2.0 ± 0.3	2.2 ± 0.2	0.023
FS (%)	39.8 ± 4.2	38.2 ± 3.1	0.088
EF (%)	71.2 ± 4.9	69.5 ± 3.7	0.094
IVSd (cm)	0.6 ± 0.1	0.6 ± 0.1	0.160
PWd (cm)	0.6 ± 0.11	0.5 ± 0.1	0.105
LVMI (g/m^2^)	68.1 ± 19.8	56.0 ± 15.1	0.005

Data are given as mean ± SD.

*EF* ejection fraction, *FS* fractional shortening, *IVSd* thickness of the interventricular septum, *LVMI* left ventricle mass index, *LVEDd* left ventricle end-diastolic diameter, *LVESd* left ventricle end-systolic diameter, *PWd* thickness of the left ventricle posterior wall, *SH* subclinical hypothyroidism.

Mitral flow E wave velocity was significantly higher in the patient group compared to the control group (*p* = 0.020). Table 3 present the pulse-wave Doppler measurements of the two groups.

**Table 3:** Pulse-wave Doppler measurements of the study groups.

	SH group (n = 35) [mean (± SD)]	Control group (n = 38) [mean (± SD)]	p value
**Left ventricle**			
Mitral E velocity (m/s)	0.88 ± 0.14	0.81 ± 0.11	0.020
Mitral A velocity (m/s)	0.54 ± 0.11	0.52 ± 0.10	0.405
Mitral E/A	1.63 ± 0.25	1.55 ± 0.25	0.171
AT (ms)	77.83 ± 14.95	84.58 ± 13.21	0.044
DT (ms)	94.29 ± 25.17	103.24 ± 16.64	0.075
ET (ms)	285.14 ± 46.26	286.89 ± 41.13	0.865
**Right ventricle**			
Tricuspid E velocity (m/s)	0.72 ± 0.11	0.69 ± 0.12	0.312
Tricuspid A velocity (m/s)	0.52 ± 0.11	0.49 ± 0.11	0.360
Tricuspid E/A	1.42 ± 0.23	1.39 ± 0.19	0.579
AT (ms)	92.83 ± 23.24	91.79 ± 19.46	0.836
DT (ms)	108.43 ± 26.85	119.76 ± 28.76	0.087
ET (ms)	278.00 ± 53.52	278.24 ± 45.54	0.984

Data are given as mean ± SD.

*AT* acceleration time, *DT* deceleration time, *ET* ejection time, *mitral A* late transmitral flow velocity, *mitral E* early transmitral flow velocity, *tricuspid A* late transtricuspid flow velocity, *tricuspid E* early transtricuspid flow velocity, *SH* subclinical hypothyroidism.

The comparison of Tissue Doppler Imaging parameters between the two groups indicated that mitral septal ejection time and deceleration time were lower (*p* = 0.003 and *p* = 0.040, respectively) and myocardial performance index was higher (*p* = 0.009) in the patient group compared to the control group. Evaluation of the right ventricular Tissue Doppler Imaging revealed that tricuspid lateral E/E_a_ and tricuspid septal E/E_a_ were higher (*p* = 0.015 and *p* = 0.024, respectively) and tricuspid septal E_a_/A_a_ and ejection time were lower (*p* = 0.018 and *p* = 0.017, respectively) in the patient group compared to the control group. Intraventricular septal A_a_ was higher (*p* = 0.022) and E_a_/A_a_ was lower (*p* = 0.032) in the patient group compared to the control group. Table 4 presents the Tissue Doppler Imaging parameters of both groups.

**Table 4:** TDI parameters of the study groups.

	SH group (n 35) [mean (± SD)]	Control group (n 38) [mean (± SD)]	p value
**Mitral lateral**			
S_a_ (m/s)	0.05 ± 0.01	0.05 ± 0.01	0.835
E_a_ (m/s)	0.11 ± 0.02	0.10 ± 0.01	0.280
A_a_ (m/s)	0.07 ± 0.02	0.06 ± 0.01	0.252
Ea/A_a_	1.68 ± 0.28	1.68 ± 0.23	0.915
IVRT (ms)	65.11 ± 17.53	64.53 ± 12.18	0.867
IVCT (ms)	58.26 ± 13.71	60.63 ± 12.43	0.440
ET (ms)	234.06 ± 25.42	244.95 ± 30.63	0.104
AT (ms)	50.77 ± 11.71	51.50 ± 11.47	0.789
DT (ms)	67.09 ± 10.73	71.00 ± 13.37	0.175
E/E_a_	8.01 ± 2.16	7.57 ± 1.65	0.331
MPI	0.53 ± 0.12	0.52 ± 0.10	0.585
**Mitral septal**			
S_a_ (m/s)	0.07 ± 0.01	0.06 ± 0.01	0.349
E_a_ (m/s)	0.11 ± 0.01	0.11 ± 0.01	0.430
A_a_ (m/s)	0.07 ± 0.02	0.06 ± 0.08	0.071
E_a_/A_a_	1.59 ± 0.30	1.67 ± 0.21	0.210
IVRT (ms)	62.51 ± 17.45	57.84 ± 10.42	0.175
IVCT (ms)	53.86 ± 11.40	52.63 ± 10.26	0.632
ET (ms)	239.26 ± 24.89	255.50 ± 19.31	0.003
AT (ms)	47.97 ± 8.83	50.89 ± 10.12	0.195
DT (ms)	69.94 ± 16.30	78.39 ± 18.10	0.040
E/A_a_	7.57 ± 1.57	7.10 ± 1.06	0.147
MPI	0.49 ± 0.11	0.43 ± 0.06	0.009
**Tricuspid lateral**			
S_a_ (m/s)	0.11 ± 0.02	0.11 ± 0.01	0.582
E_a_ (m/s)	0.15 ± 0.02	0.15 ± 0.02	0.242
A_a_ (m/s)	0.11 ± 0.03	0.11 ± 0.02	0.574
E_a_/A_a_	1.40 ± 0.30	1.49 ± 0.26	0.202
IVRT (ms)	60.43 ± 10.02	57.32 ± 11.41	0.339
IVCT (ms)	60.77 ± 14.60	59.11 ± 11.25	0.585
ET (ms)	246.43 ± 35.33	249.71 ± 23.12	0.643
AT (ms)	65.69 ± 15.86	70.87 ± 16.41	0.135
DT (ms)	82.74 ± 22.45	91.71 ± 21.09	0.083
E/E_a_	4.92 ± 0.97	4.40 ± 0.80	0.015
MPI	0.50 ± 0.13	0.46 ± 0.08	0.154
**Tricuspid septal**			
S_a_ (m/s)	0.08 ± 0.01	0.07 ± 0.01	0.262
E_a_ (m/s)	0.11 ± 0.02	0.12 ± 0.01	0.245
A_a_ (m/s)	0.08 ± 0.02	0.07 ± 0.02	0.520
E_a_/A_a_	1.54 ± 0.30	1.69 ± 0.24	0.018
IVRT (ms)	57.94 ± 16.13	55.55 ± 8.97	0.443
IVCT (ms)	52.26 ± 10.03	53.68 ± 10.01	0.545
ET (ms)	242.17 ± 26.80	255.42 ± 19.37	0.017
AT (ms)	51.97 ± 13.19	54.50 ± 6.96	0.304
DT (ms)	70.63 ± 17.43	75.53 ± 12.36	0.174
E/E_a_	6.30 ± 1.18	5.67 ± 1.14	0.024
MPI	0.46 ± 0.10	0.43 ± 0.05	0.156
**Interventricular septum**			
S_a_ (m/s)	0.05 ± 0.00	0.05 ± 0.01	0.070
E_a_ (m/s)	0.10 ± 0.02	0.10 ± 0.01	0.722
A_a_ (m/s)	0.06 ± 0.01	0.05 ± 0.00	0.022
E_a_/A_a_	1.76 ± 0.31	1.90 ± 0.23	0.032
IVRT (ms)	57.80 ± 14.53	57.68 ± 13.32	0.972
IVCT (ms)	53.06 ± 11.36	55.13 ± 9.58	0.401
ET (ms)	244.63 ± 23.39	251.26 ± 18.88	0.185
AT (ms)	52.97 ± 9.38	56.00 ± 9.13	0.167
DT (ms)	55.54 ± 8.46	60.97 ± 11.23	0.023
MPI	0.46 ± 0.08	0.44 ± 0.07	0.379

Data are given as mean ± SD.

*A*_*a*_ atrial diastolic wave, *AT* acceleration time, *DT* deceleration time, *E* early transvalvular flow velocity, *E*_*a*_ early diastolic wave, *ET* ejection time, *IVCT* isovolumic contraction time, *IVRT* isovolumic relaxation time, *MPI* myocardial performance index, *S*_*a*_ myocardial systolic wave, *SH* subclinical hypothyroidism, *TDI* tissue Doppler imaging.

Serum thyroid stimulating hormone level established a correlation with two parameters; a positive correlation was established with mitral septal Aa (r =  + 0.380, p = 0.024) and a negative correlation was established with mitral flow ejection time (r = − 0.456, p = 0.006).

## Discussion

Myocardial tissue is highly sensitive to thyroid hormones due to its excess of thyroid hormone receptors^18^. Hypothyroidism could trigger cardiac remodeling in 3 manners. First, hypothyroidism reduces activity of several enzymes involved in intracellular calcium handling, thereby leading to alteration in the expression of contractile protein. Second, cardiac dysfunction may be caused by the chronic inflammation and tissue changes induced by subclinical hypothyroidism. Third, subclinical hypothyroidism is responsible for the hemodynamic alterations associated with cardiac impairment^18–20^. As a result of these, hypothyroidism leads to numerous changes in cardiac contractile, myocardial oxygen use, stroke volume, blood pressure, and systemic vascular resistance^21^. The effects of subclinical hypothyroidism on cardiac functions and Tissue Doppler Imaging parameters have mostly been reported in adult studies^10,11,15,16,18,22–24^. Although the number of studies reporting on the cardiac effects of subclinical hypothyroidism have been growing recently, particularly in children, there is still scarcity in the number of studies conducted in children^9,25^.

Left ventricle mass has been shown to increase in adults with subclinical hypothyroidism^11,18^. Rodonti et al.^12^ reported that left ventricular mass might increase in patients with thyroid stimulating hormone > 10 mU/l during long-term follow-up although no increase might be seen at baseline measurements. A review of the English-language literature showed that there have been only two studies reporting on increased left ventricular mass in children with subclinical hypothyroidism^9,26^. Of these, the study by Catli et al.^9^ evaluated 31 children with subclinical hypothyroidism and found a significant increase in left ventricular mass index and interventricular septal thickness. The other study was conducted by Sert et al.^26^ which evaluated obese children with subclinical hypothyroidism and found increased left ventricular mass index in children with thyroid stimulating hormone > 4 mIU/L compared to children with thyroid stimulating hormone < 4 mIU/L. Therefore, the present study is of prime value since it is one of the few studies in the literature showing that left ventricular mass index significantly increases in children with subclinical hypothyroidism.

Studies investigating mitral and tricuspid flow pulsed-wave Doppler have indicated that diastolic dysfunction may develop in children with subclinical hypothyroidism. Irdem et al.^25^ found that mitral E-wave velocity and E/A ratio were lower and mitral A-wave and IVRT were higher in children with subclinical hypothyroidism compared to healthy controls. Another study showed that no differences were found in mitral flow diastolic parameters (mitral flow E velocity, A velocity, E/A ratio, and deceleration time) between patients with subclinical hypothyroidism and healthy controls^9^. Some adult studies have also indicated that left ventricular systolic, in particular, and diastolic dysfunction may occur in patients with subclinical hypothyroidism^15,22–24^. In our study, mitral flow E velocity was higher in children with subclinical hypothyroidism compared to healthy controls, which contradicted with the findings of other studies that found lower mitral flow E velocity in children with subclinical hypothyroidism compared to controls^9,25^. However, a previous study that evaluated adults with subclinical hypothyroidism reported that peak E velocity was markedly higher in patients with thyroid stimulating hormone > 10 mU/L and that this finding represented left atrial pressure and was also associated with heart failure in patients with thyroid stimulating hormone > 10 mU/L^11^.

Subclinical hypothyroidism has been commonly associated with Tissue Doppler Imaging in adult studies^10,16,18,23^. However, this association has been scarcely reported in pediatric studies^9,25^. Catli et al.^9^ reported that subclinical hypothyroidism was associated with left ventricular diastolic (lower E′_m,_ higher E/E′_m_ ratio, longer IVRT) and systolic (lower IVCT) dysfunctions in children with subclinical hypothyroidism. In addition, the study also found that lateral mitral myocardial performance index was higher, systolic IVCT was higher and ejection time was lower, and diastolic IVRT and myocardial performance index were higher. In contrast, Irdem et al.^25^ revealed that left ventricular septal E_m_ velocity and E_m_/A_m_ ratio were markedly decreased and A_m_ velocity was markedly increased in children with subclinical hypothyroidism compared to healthy controls. The authors also reported that left ventricular lateral E_m_ velocity and E_m_/A_m_ ratio were lower and E/E_m_ ratio was higher in the patient group.

Myocardial performance index is a simple method used in the evaluation of systolic and diastolic cardiac functions. Moreover, increased myocardial performance index is an indicator of impaired systolic and diastolic functions^27^. In the present study, increased mitral septal myocardial performance index and ejection time indicated impaired diastolic and global ventricular function. Catli et al.^9^ found increased mitral lateral myocardial performance index in children with subclinical hypothyroidism, whereas we found increased mitral septal myocardial performance index. These two findings suggest that global ventricular function may be impaired in children with subclinical hypothyroidism.

Besides left ventricular functions, right ventricular functions may also be impaired in patients with subclinical hypothyroidism^10,16,18^. In our study, of the right ventricular Tissue Doppler Imaging diastolic parameters analyzed, tricuspid septal and lateral E/E_a_ ratio were higher in children with subclinical hypothyroidism compared to controls, which indicated right ventricular diastolic dysfunction (impaired ventricular filling). Catli et al.^9^ reported that mitral annulus E/A_a_ ratio was higher in children with subclinical hypothyroidism compared to controls; however, we found no study in the literature reporting on increased tricuspid annulus E/A_a_ ratio in children with subclinical hypothyroidism. On the other hand, in our study, we found that tricuspid septal E_a_/A_a_ was lower in children with subclinical hypothyroidism compared to controls. A similar finding was reported by Kosar et al.^16^, who suggested that this finding was another indicator of right ventricular diastolic dysfunction. The same study also revealed that both mitral annular E_a_/A_a_ ratio and tricuspid annular E_a_/A_a_ ratio were lower and a negative correlation was found between thyroid stimulating hormone and E_a_ velocity and E_a_/A_a_ ratio. The findings reported by Kosar et al.^16^ suggest that not only left ventricular diastolic functions but also right ventricular diastolic functions may be impaired in patients with subclinical hypothyroidism. Similarly, we also found that tricuspid septal ejection time was lower in children with subclinical hypothyroidism compared to controls, which indicated impairment in right ventricular systolic functions. In this regard, our study is the first study in the literature showing that left ventricular dysfunction as well as right ventricular systolic and diastolic dysfunction may occur in children with subclinical hypothyroidism.

Increased A_a_ wave and decreased E_a_/A_a_ ratio at interventricular septum may indicate impaired global ventricular diastolic functions. Catli et al.^9^ also investigated interventricular septum Tissue Doppler Imaging and suggested that systolic (higher IVCT, lower ejection time), diastolic (higher IVRT), and global (higher myocardial performance index) functions may be impaired in children with subclinical hypothyroidism.

There are adult studies showing that some myocardial dysfunction indicators measured by Tissue Doppler Imaging and conventional echocardiography have significant correlations with serum thyroid stimulating hormone levels in subclinical hypothyroidism^10,18^. Our study revealed that as thyroid stimulating hormone level increases, mitral flow ejection time decreases (related to left ventricular systolic dysfunction) and mitral septal Aa increases (related to left ventricular diastolic dysfunction). This correlation indicates the importance of the effect of increased thyroid stimulating hormone level on the development of left ventricular systolic and diastolic dysfunction in children with subclinical hypothyroidism. To our knowledge, this correlation has never been reported in any study in the literature. In our study, thyroid stimulating hormone levels were significantly different between the subclinical hypothyroidism and the control group; however, sT4 and sT3 levels were similar, and no significant correlation was found between sT4 and sT3 levels and myocardial dysfunction indicators as in TSH. For these reasons, we think that the main cause of myocardial dysfunction is thyroid stimulating hormone.

## Conclusion

Subclinical hypothyroidism may lead to increased left ventricular mass index. Our study is one of the few studies in the literature showing that left ventricular mass index may be increased in children with subclinical hypothyroidism. Although the present study showed that increased left ventricular mass index may have adverse effects on ventricular functions in children with subclinical hypothyroidism, further studies with longer periods are needed to investigate these effects. Our study also indicated that left ventricular systolic and diastolic Tissue Doppler Imaging parameters (lower mitral septal ejection time, higher mitral septal myocardial performance index) as well as right ventricular systolic (lower tricuspid septal ejection time) and diastolic (higher tricuspid septal and lateral E/E_a_, lower tricuspid septal E_a_/E_a_) functions may be impaired in children with subclinical hypothyroidism. Although left and right ventricular dysfunctions have been commonly reported in adults with subclinical hypothyroidism in echocardiographic studies, our study is the first study in the literature showing that left ventricular dysfunction as well as right ventricular systolic and diastolic dysfunction may occur in children with subclinical hypothyroidism.

### Study limitations

This study has several limitations. Firstly, the study evaluates cardiac functions in children with SH. However, long-term follow-up of patients and cardiac functions have not been performed. For this reason, longer-term studies are needed. The small number of patients may also act as another limitation of our study.
